# Effect of levothyroxine on cardiac function in children with subclinical hypothyroidism: A quasi-experimental study

**DOI:** 10.22088/cjim.10.3.332

**Published:** 2019

**Authors:** Nasrin Banu Rupani, Morteza Alijanpour, Kazem Babazadeh, Karimollah Hajian-Tilaki, Fatemeh Moadabdoost

**Affiliations:** 1 Student Research Committee, School of Medicine, Babol University of Medical Sciences, Babol, Iran; 2 Non-Communicable Pediatric Diseases Research Center, Health Research Institute, Babol University of Medical Sciences, Babol, Iran; 3 Babol University of Medical Sciences, Babol, Iran; 4 The Clinical Research Development Unit of Amirkola Children's Hospital, Babol University of Medical Sciences, Babol, Iran

**Keywords:** Hypothyroidism, Child, Cardiac function, Levothyroxine

## Abstract

**Background:**

Because of the importance of thyroid function and its effect on different organs, such as the heart, this study was aimed to evaluate the effect of levothyroxine on cardiac function in children with subclinical hypothyroidism.

**Methods:**

This quasi-experimental study was performed on children aged 4-12 years old with diagnosis of subclinical hypothyroidism in Amirkola Children's Hospital during 2018-2019. Cardiac functional parameters, including ejection fraction (EF), fractional shortening (FS), ratio of early filling velocity to early diastolic mitral annulus velocity (E/E'), myocardial performance index (MPI), left ventricular end-diastolic diameter (LVEDD), and left ventricular end-systolic diameter (LVESD), were measured by echocardiography at baseline and 6 months after levothyroxine treatment.

**Results:**

Out of the 30 subjects, 19 (63.3%) were boys and 11 (36.7%) were girls. The mean age was 6.60±2.13 years old. The mean EF index was 63.13±3.01 percent before treatment, which increased to 69.07±4.50 percent after treatment (p<0.001). Also, the mean FS was 31.83±1.62 percent before treatment, which improved to 35.10±1.13 percent after treatment (p<0.001). The mean MPI was 0.28±0.02 before treatment, which increased to 0.33±0.03 after treatment (p<0.001). On the other hand, no significant difference was found in the mean E/E' before and after treatment. The mean LVEDD decreased from 3.47±0.46 cm before treatment to 3.05±0.40 cm after treatment (p<0.001), whereas LVESD non-significantly decreased after treatment versus before treatment.

**Conclusion:**

The results showed that treatment with levothyroxine may improve cardiac functional parameters in children with subclinical hypothyroidism.

Subclinical hypothyroidism is a branch of hypothyroidism which is characterized by increased thyroid stimulating hormone (TSH) and normal thyroxine (T4) (1). The prevalence of subclinical hyperthyroidism in pediatrics is about 2% and its incidence is more in girls than in boys (2, 3). Some of the complications of subclinical hypothyroidism include changing lipid profiles, increase blood coagulation and changing cardiac function especially left ventricle (4, 5). Cardiovascular system is sensitive to thyroid hormone activity. Although a wide spectrum of cardiac disorders has been identified in patients with thyroid dysfunctions, cardiac involvement in patients with subclinical hypothyroidism has been discussed over the recent two-three decades. Effects of subclinical hypothyroidism on cardiovascular system are directly related to severity of thyroid hormone deficiency. Subclinical hypothyroidism causes increase in systemic vascular resistance, diastolic dysfunction and decrease in preload of heart (6, 7). It can also lead to heart failure and coronary artery lesions (8).

There is conflicting evidence on the effect of subclinical hypothyroidism on decrease in systolic functions. Some papers stated that subclinical hypothyroidism can decrease systolic function (9, 10), but some others are not compatible with this result (11, 12). Thyroid hormones can affect cardiovascular system through transcriptional regulation and regulatory proteins (13, 14). In addition, hypothyroidism can contribute in chronic inflammations and tissue changes (such as dehydration and collagen alternation), and also hemodynamic changes by influence on arterial smooth muscle (15, 16). Cardiovascular complications of subclinical hypothyroidism can be seen in children and adults.

Some articles stated that treatment with levothyroxine was associated with positive effects on cardiac function in adults with subclinical hypothyroidism suffering from cardiovascular complications (17-20). However, number of studies assessing the effects of levothyroxine on cardiac complications in pediatric population is limited. Considering the importance of proper thyroid function and its effect on different organs, such as heart which has a vital role in correct function of various organs and in improving the growth and development of children, it would be clinically helpful to find a therapeutic option in pediatric patients with subclinical hypothyroidism to treat and prevent them from potential cardiac complications in the future. The purpose of the present study was to evaluate the effects of treatment with levothyroxine on cardiac function in the pediatric patients with subclinical hypothyroidism.

## Methods

This open-label quasi-experimental study was performed in 2018-2019 on children aged 4-12 years old, who were referred to the endocrinology clinic of the Amirkola Children's Hospital with the diagnosis of subclinical hypothyroidism, or the children who came for growth evaluation and were diagnosed as subclinical hypothyroidism by laboratory tests (elevated TSH and normal T4). Diagnosis of subclinical hypothyroidism was done by pediatric endocrinologist.

The exclusion criteria were as follows:

1. Being overweight (body mass index [BMI, kg/m^2^] between 85^th^ and 95^th^ percentile), obesity (BMI higher than 95^th^ percentile), or underweight (BMI lower than 5^th^ percentile) (21).
2. Hypertension (higher than 95^th^ percentile for age, sex and height) (21)
3. Previous use of levothyroxine, anticonvulsants
4. Precocious puberty (22, 23)
5. Pathologic bradycardia or tachycardia (24)
6. Congenital or acquired heart disease and under medical treatment
7. Liver or kidney disease
8. Hyperlipidemia
9. Diabetes
10. Infectious diseases

All subjects were initially examined for thyroid, maturity stage, hypertension and pulse rate and their height and weight were recorded. The examination was conducted by the pediatric resident under supervision of the pediatric endocrinologist. Height and weight of the patients were evaluated and recorded by BALAS equipment (Nikan Andishan Nozhan Co, Tehran, Iran) with measurement error of 0.2 grams and then BMI was calculated using formula of weight (kilograms) divided by height (square meters). Blood pressure was assessed in the prone and rest position using the sphygmomanometer Heine (made in Germany). To conduct thyroid tests, 2 cc blood was collected after 8-10 hours fasting and then levels of TSH and T4 were measured using enzyme-linked immunosorbent assay (ELISA) kits (Pishtaz Teb, Tehran, Iran) in the laboratory of Amirkola Children's Hospital. Normal range for THS was 0.5-5.5 mIu/l and for T4 was 5.5-12.8 µg/dl (prepubertal children: 3-10 years old) or 4.2-13µg/dl (pubertal children >10 years old) (24).

After approving subclinical hypothyroidism, the subjects underwent echocardiography for the evaluation of cardiac function, using a Medison Accuvix V10 ultrasound equipment (Samsung Medison, Seoul, South Korea) by the pediatric cardiologist. Study outcomes and parameters of cardiac function assessed were as follows:

- **Left ventricular end-systolic diameter (LVESD)**: Normal value depends on age, including 1.9±0.3 (1-5 years old), 2.4±0.3 (6-9 years old), 2.7±0.3 (10-13 years old) (25).
- **Left ventricular end-diastolic diameter (** **LVEDD** **)**: Normal value depends on age, including 3.1±0.4 (1-5 years old), 3.9±0.4 (6-9 years old), 4.3±0.4 (10-13 years old) (25).
- **Fractional shortening**: It is defined as percentage of changes of left ventricular dimensions between end-diastole and end-systole (25). It is measured using the following formula and the normal range is between 28% and 38%: (LVEDD-LVESD)/LVEDD×100.
- **Ejection fraction (EF)**: It is a volume parameter showing changes of left ventricular volume between end-diastole and end-systole (25). It is measured using the following formula and the normal range is between 54% and 75%: (left ventricular end-diastolic volume [LVEDD]- left ventricular end-systolic volume [LVESD])/LVEDD×100.
- **Ratio of early filling velocity** **to early diastolic mitral annulus velocity (E/E')**: It is a strong predictor of post-acute myocardial infarction complications, hypertensive cardiac diseases, severe secondary mitral insufficiency, end stage renal disease, atrial fibrillation and cardiomyopathies (26). Normal value depends on age, including 8.1±1.8 (1-5 years old), 7.7±1.6 (6-9 years old), 6.6±1.4 (10-13 years old).

- **Myocardial performance index** **(MPI) or** **Tei index**: It includes both systolic and diastolic time intervals to assess the global cardiac dysfunction (26). It is measured using the following formula and the normal value is 0.35±0.03 for left ventricle: (Isovolumic relaxation time+Isovolumic contraction time)/ Ejection time.

Patients with abnormal range of the mentioned parameters and clinical symptoms of heart failure, such as fatigue, anorexia, cough, dyspnea, sweating, abdominal pain, edema, hepatomegaly, orthopnea, rale in auscultation, and so on, were excluded from the study. The sample size was estimated as 30 patients per group by a power of 80%, effect size of 0.7 and confidence level of 95%. Treatment was started with tablet levothyroxine 0.1 mg (Iran Hormone Pharmaceutical Co, Tehran, Iran) with dose of 4 µ/kg/day before breakfast. Echocardiography was performed before and 6 months after starting levothyroxine treatment by the same cardiologist. The cardiologist was unaware of the intervention groups. Thyroid function tests (TSH and T4) were reassess 1.5 months after starting treatment and then every 2 months. 

The obtained data were analyzed using SPSS statistical software. The descriptive analysis was used for the determination of the frequency, percentages, mean and standard deviation. Normality of the data was tested using the Kolmogorov-Smirnov test. Paired t-test and Wilcoxon signed rank were used to compare the parametric and non-parametric data, respectively, before and after the treatment. We utilized the exact method of bootstrap to calculate the p-value. A p-value of <0.05 was considered to be significant in all tests. The informed written consent was signed by all subjects' parents at the beginning of the study. The patients’ information was kept confidential. This study was approved by the Ethics Research Committee of Babol University of Medical Sciences (code: IR.MUBABOL.HRI.REC.1397.153). This trial was registered in the Iranian Registry of Clinical Trials with the number IRCT20180228038900N3.

## Results

**Figure 1:**
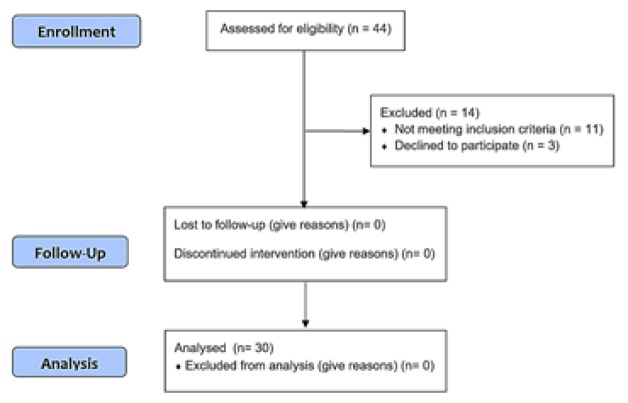
CONSORT flow diagram

Out of 30 subjects, 19 (63.3%) were boys and the rest were girls. The mean serum level of T4 was 8.42±2.01 µg/dl before treatment and 8.38±2.22 µg/dl at six months of treatment (p=0.86). The mean serum level of TSH was 8.77±1.47 mIu/l before treatment and 3.36±2.17 mIu/l at six months of treatment (<0.001). Figure 1 indicates the CONSORT flow diagram. Table 1 also shows the baseline information of the patients.

**Table 1:** Baseline information of children with subclinical hypothyroidism

**Variable**	**Results**
Sex (boy/girl), number	19/11
Age, mean±SD, years old	6.60±2.13
Body Mass Index, mean±SD, kg/m^2^	16.55±4.00
T4, mean±SD, µg/dl	8.42±2.01
TSH, mean±SD, mIu/l	8.77±1.47

Table 2 shows comparison of cardiac functional parameters before and six months after treatment with levothyroxine in the patients. The mean EF index was 63.13±3.01 before treatment, which increased to 69.07±4.50 after treatment (p<0.001). Also, the mean FS was 31.83±1.62 before treatment, which improved to 35.10±1.13 after treatment (p<0.001). The mean MPI was 0.28±0.02 before treatment, which increased to 0.33±0.03 at six months of treatment (p<0.001). On the other hand, no significant difference was found in the mean E/E' before and after treatment. The mean LVEDD decreased from 3.47±0.46 before treatment to 3.05±0.40 after treatment (p<0.001), whereas LVESD did not significantly decrease after treatment compared with before treatment. Table 3 indicates comparison of cardiac functional parameters before and six months after treatment with levothyroxine by sex. In both boys and girls, EF, FS and MPI significantly increased after treatment compared with before treatment. LVEDD also significantly decreased after treatment compared with before treatment in both sexes. E/E' parameter increased in boys, but did not significantly increase in girls.

**Table 2:** Cardiac functional parameters before and six months after treatment with levothyroxine, in children with subclinical hypothyroidism

**Variables**	**Before treatment ** **(mean±SD)**	**Six months of treatment ** **(mean±SD)**	**P-value**
Systolic parameters			
EF^a^ (%)	63.13±3.01	69.07±4.50	<0.001
FS^b^ (%)	31.83±1.62	35.10±1.13	<0.001
Diastolic parameter			
E/E'^c^	6.31±0.71	6.49±0.74	0.07
Global ventricular performance			
MPI^d^	0.28±0.02	0.33±0.03	<0.001
Cardiac morphological parameters			
LVEDD^e^ (cm)	3.47±0.46	3.05±0.40	<0.001
LVESD^f^ (cm)	2.08±0.36	2.02±0.44	0.64

a Ejection fraction;

b Fractional shortening;

c Ratio of early filling velocity to early diastolic mitral annulus velocity;

d myocardial performance index;

e left ventricular end-diastolic diameter;

f left ventricular end-systolic diameter

**Table 3:** Cardiac functional parameters before and six months after treatment with levothyroxine, in children with subclinical hypothyroidism by sex

**Sex**	**Variables**	**Before treatment ** **(mean±SD)**	**Six months of treatment ** **(mean±SD)**	**P-value**
Boys	Systolic parameters			
	EF^a^ (%)	62.95±3.29	69.63±4.17	<0.001
	FS^b^ (%)	31.42±1.50	35.05±2.19	<0.001
	Diastolic parameter			
	E/E'^c^	6.34±0.63	6.60±0.72	0.010
	Global ventricular performance			
	MPI^d^	0.28±0.03	0.33±0.02	<0.001
	Cardiac morphological parameters			
	LVEDD^e^ (cm)	3.49±0.48	3.01±0.45	<0.001
	LVESD^f^ (cm)	2.09±0.30	2.00±0.48	0.990
Girls	Systolic parameters			
	EF (%)	63.45±2.52	68.09±5.06	0.040
	FS (%)	32.55±1.63	35.18±2.13	0.010
	Diastolic parameter			
	E/E'	6.27±0.87	6.31±0.76	0.84
	Global ventricular performance			
	MPI	0.28±0.01	0.33±0.04	0.003
	Cardiac morphological parameters			
	LVEDD (cm)	3.42±0.45	3.12±0.30	0.006
	LVESD (cm)	2.06±0.17	2.05±0.39	0.380

a Ejection fraction;

b Fractional shortening;

c Ratio of early filling velocity to early diastolic mitral annulus velocity;

d myocardial performance index;

e left ventricular end-diastolic diameter;

f left ventricular end-systolic diameter

## Discussion

To our knowledge, this is the first study in Iran that investigated the effects of levothyroxine on cardiac function in children with subclinical hypothyroidism using systolic, diastolic and morphologic parameters. As observed, systolic parameters improved after treatment. In addition, treatment with levothyroxine had positive effects on MPI and LVEDD.

So far, limited data has been published similar to our topic. In one study by Çatlı et al. (27) in Turkey, which was performed on 31 children with subclinical hypothyroidism, it was seen that the morphological parameters of LVEDD and LVESD increased in 6 months of treatment that the first result was in agreement with our finding, but the latter was not compatible with our data. Çatlı et al. (27) also mentioned that other cardiac functional parameters, such as EF, FS and MPI did not significantly change after 6 months, which were contrary to our results. The authors stated that short duration of treatment might be a reason for lack of changes. The incompatibility between their results and ours might be due to differences in age, BMI, and/or serum levels of TSH and T4. Adult studies did not only showe that patients with subclinical hypothyroidism had impaired myocardial contractility and diastolic dysfunction, but also stated that these problems may be reversed with levothyroxine treatment (28-31). The study by Nakova et al. (10) on 54 patients showed that a 5-month levothyroxine treatment could contribute to higher EF and lower E/E'. Another research by Tadic et al. (32) on 54 Serbian women with subclinical hypothyroidism indicated that parameter of E/E' improved after 1-year treatment, but no changes were found in LVEDD and LVESD. The last results were compatible with the article by Ilic et al. (33), in which they reported that no significant changes were seen in LVEDD and LVESD after 1-year treatment with levothyroxine. A large-scale cohort study by Andersen et al. (34) revealed that levothyroxine treatment did not reduce risk of mortality in patients with subclinical hypothyroidism and heart disease. These data are helpful for clinicians to select a preventive and therapeutic approach against cardiac complications of subclinical hypothyroidism through levothyroxine administration, however, stronger evidence is needed in this regard. We also evaluated the effect of levothyroxine by sex. The notable point found from the results was improvement of E/E' parameter in boys but not in girls after treatment. This may be explained by differences in hormonal profiles between sexes. To the best of our knowledge, except Çatlı et al.' study, there are no other children studies to compare our findings with. Considering that the frequency of children with subclinical hypothyroidism is not low and they impose high economic costs on families and societies, addition of cardiac compactions following subclinical hypothyroidism increase pressure on children and their family. Therefore, more studies are suggested to be performed on children and adolescent patients with subclinical hypothyroidism. These studies can increase awareness and information about therapeutic drugs and help clinicians for better management of the patients.

In most of the papers, it has been exhibited that subclinical hypothyroidism is related to different cardiac functional parameters (35, 36). Studies on patients with subclinical hypothyroidism, who underwent levothyroxine treatment, showed evidence of improving left ventricular systolic function. Therefore, subclinical hypothyroidism can be considered as a mild hypothyroidism which is associated with primary cardiovascular symptoms. Thus, starting early and timely treatment in these patients seems necessary to prevent cardiac involvement.

One of the limitations of our study was the small sample size and relatively short follow-up period of study participants. Second, our study did not have an untreated control group (e.g., including patients who did not agree to undergo levothyroxine treatment) and we cannot certainly and strongly say that the observed improvements of cardiac function were a result of the levothyroxine treatment and not other factors, like physiological growth. Overall, it has been proposed to design new studies with bigger sample size, longer follow-up period and an untreated control group, plus assess other cardiac functional indices, such as left ventricle mass index, thickness of the left ventricle posterior wall, and mitral deceleration time.

In conclusion, according to the results, it can be concluded that treatment with levothyroxine may improve cardiac functional parameters in the children with subclinical hypothyroidism. However, it is necessary to perform new studies with an untreated control group to confirm the positive effect of levothyroxine on cardiac functions in childhood subclinical hypothyroidism.
